# Experimental Warming Hastens Physical Dormancy Break and Germination in Tropical Fabaceae

**DOI:** 10.3389/fpls.2021.782706

**Published:** 2021-12-15

**Authors:** Ganesh K. Jaganathan, Matthew Biddick

**Affiliations:** ^1^Department of Biothermal Engineering, University of Shanghai for Science and Technology, Shanghai, China; ^2^Terrestrial Ecology Research Group, Technical University of Munich, Freising, Germany

**Keywords:** climate change, germination ecology, impermeable seed coat, soil seed banks, soil temperature

## Abstract

Climate warming may threaten the germination strategies of many plants that are uniquely adapted to today’s climate. For instance, species that employ physical dormancy (PY) – the production of seeds that are impermeable to water until high temperatures break them, consequently synchronizing germination with favorable growing conditions – may find that their seeds germinate during unfavorable or potentially fatal periods if threshold temperatures are reached earlier in the year. To explore this, we subjected the seeds of five species with physical dormancy (from the genera *Abrus*, *Bauhinia*, *Cassia*, *Albizia*, and *Acacia*) to “mild” (+2°C) and “extreme” (+4°C) future warming scenarios and documented their germination over 2 years relative to a control treatment. Under current climatic conditions, a proportion of seeds from all five species remained dormant in the soil for 2 years. A mild warming of 2°C had little to no effect on the germination of four of the five study species. Contrastingly, an extreme warming of 4°C dramatically increased germination in all five species within the first year, indicating a reduction in their ability to persist in the soil long-term. *Cassia fistula* was particularly susceptible to warming, exhibiting a similar increase in germination under both mild and extreme warming relative to control. Our findings suggest that climate warming in the tropics may cause the seeds of species that rely on physical dormancy to stagger the risk of unsuccessful germination across years to leave soil seed banks prematurely – the long-term implications of which remain unknown.

## Introduction

The life cycle of plants is intricately linked to the climate. Climate change, therefore, may threaten life history strategies that have arisen over evolutionary timescales. Understanding how plant communities will respond to anthropogenic climate change has become an integral part of ecological research (Warren et al., 2013). Seeds are particularly useful in this endeavor as they enable us to evaluate changes in vegetation at the community level (Walck et al., 2011). Many species have evolved dormancy mechanisms that regulate when germination takes place – an adaptation that enables plants to inhabit environments with volatile climates (Finch-Savage and Leubner-Metzger, 2006; Willis et al., 2014). However, the warming of Earth’s climate could cause seeds to germinate at unfavorable times for seedling establishment, potentially leading to local extinctions. Unfortunately, our understanding of how a warmer climate will affect the germination ecology of species with dormant seeds is hampered by a lack of long-term studies.

Seed dormancy can be advantageous in the tropics, where synchronizing germination with the rainy season ensures seedlings are well established before the onset of the harsh dry season (Garwood, 1983; Khurana and Singh, 2001). Tropical plants often avoid dry season germination by either producing dormant seeds or dispersing their seeds in the wet season (Sautu et al., 2007; Salazar et al., 2011; Ramos et al., 2017; Escobar et al., 2018). Thus a trade-off exists, whereby species that disperse seeds during the wet season tend to be non-dormant, germinating immediately following dispersal, while species that disperse their seeds in the dry season tend to be dormant, germinating only when conditions become favorable again in the wet season (Salazar et al., 2011; de Souza et al., 2020). Of the latter, various strategies are used to achieve seed dormancy.

Physical dormancy (PY), caused by a seed/fruit coat that is impermeable to water in some genera of only 18 contemporary angiosperm families is found in 25% of all flowering plants (Baskin and Baskin, 2004; Hudson et al., 2015). It is the second most common class of dormancy after physiological dormancy (PD) and is achieved late in seed development when seeds dry below threshold moisture content (Barrett-Lennard and Gladstones, 1964; Geneve, 2009; Jaganathan, 2016). Impermeable seeds persist in the soil until specific environmental conditions stimulate dormancy-break by opening structures on the seed coat known as “water-gaps” through which water enters and initiates germination (Van Assche et al., 2003; Baskin and Baskin, 2004). PY is typically broken by temperature fluctuations prior to the growing season (Cook et al., 2008; Jayasuriya et al., 2009; Rodrigues-Junior et al., 2018), however, it can also be broken by erratic events such as fire (Jaganathan, 2015) or passage through an animal gut (Jaganathan et al., 2016; Milotić and Hoffmann, 2016). PY can benefit plant fitness not only by synchronizing germination with favorable growing conditions (Baskin et al., 2000), but also by establishing a long-term soil seed bank from which only a portion germinate each year, spreading germination risk across years (Baskin and Baskin, 2014), and protecting seeds against pathogens, soil microbes (Dalling et al., 2011), and seed predators (Paulsen et al., 2013).

While global mean annual temperature is predicted certainly to rise, e.g., 4°C by 2100 (Warren et al., 2013), consequences this change will have for species that employ seed dormancy remain poorly understood. Much of our knowledge of how a warmer climate affects seed dynamics comes from studies conducted in alpine ecosystems (Mondoni et al., 2012; Hoyle et al., 2013); presumably, because they are disproportionately vulnerable to changes in temperature. Tropical ecosystems, on the other hand, have received comparatively less attention (Perez et al., 2016; Stroud and Feeley, 2017). This is partly due to disagreement over the extent to which temperature will rise in the tropics, with predictions ranging widely from 0.26 to 5°C (Corlett, 2012). Regardless of extent, consensus is emerging that warming in the tropics could have serious consequences for plant life (Feeley and Silman, 2010). Soil temperature acts as a bottleneck that controls, not only when seeds break dormancy, but also the proportion of seeds germinating each year. Climate warming may therefore alter the soil environment in which the seeds are present following dispersal. This may lead to increased mortality *via* the excess evaporation of soil water (Ooi, 2012) or the stimulation of dormancy-break (and consequently germination) at unfavorable times, such that seedlings die before establishing (Walck et al., 2011).

Seeds banks are vital to species that inhabit ecosystems with unpredictable climates or short growing seasons (Long et al., 2015; Jaganathan et al., 2019). They can enable species to persist locally, even when seemingly all plant life has been destroyed (Jaganathan et al., 2015). However, while it is becoming increasingly apparent that warming temperatures may significantly affect the regeneration ecology of species with seed dormancy (Orru et al., 2012; Newton et al., 2020), responses appear to be largely species-specific (Seal et al., 2017). Further, despite efforts to understand such effects for species that employ PY (Hudson et al., 2015; Cochrane, 2017), to date, no such study has been done in the tropics. To close this gap, we performed a long-term warming experiment to investigate how a warmer climate will affect the germination ecology of five tropical species with PY. Specifically, we asked:

1. How will climate warming in the tropics affect the germination ecology of species with PY?
2. Do effects differ between mild and extreme warming scenarios?
3. Are responses to climate warming species-specific?

## Materials and Methods

### Study Site

This study was conducted in the Western Ghats of southern India, which is one of the 100 biodiversity hot-spots identified by Myers et al. (2000). The Western Ghats, a discontinuous chain of mountains located on the western side of the Indian peninsular, covers an area of 140,000 km^2^ that extends from the Tapti river valley in Gujarat to Kanyakumari in Tamil Nadu. The mountain range is approximately 1,600 km long (Nayar et al., 2014) and has some 8,080 species of flowering plants, of which 1,273 are endemic. The Indian Ministry of Environment and Forestry (MoEF) has estimated that the temperature in the Western Ghats has increased by 1.7–1.8°C since the 1970’s and predicts a further increase of 3–4°C before the end of this century (Sharma and Chauhan, 2011). Our understanding of how this warming will affect the Western Ghats region is hindered by the fact that many of the native plant species are long-lived and are therefore slow to exhibit any changes.

### Study Species and Seed Collection

We selected the following five species namely *Abrus precatorius*, *Bauhinia tomentosa*, *Cassia fistula*, *Albizia lebbeck*, and *Acacia chundra*, that occur abundantly in the Western Ghats of India but are also distributed across other tropical and sub-tropical regions (see Table 1). We selected these species because of their abundance and wide distribution. Understanding the impacts of climate warming on the germination ecology of these species can therefore help inform conservation strategies for not only these five species but also related or ecologically similar taxa. Furthermore, prolific species can serve as models to estimate how other endangered species might be affected.

**Table 1:** The sub-family, collection date, location, moisture content at the time of collection (average ± S.D.), percentage of permeable seeds, and distribution and life form of the five legume species studied.

Species	Sub-family	Collection date	Location	Moisture content (%)	% of permeable seeds at the time of collection	Distribution	Life form
*Abrus precatorius*	Papilionoideae	10/01/2017	11° 5' 46.4712" N 76° 45' 39.2688" E	7.57 ± 3.02	20	Temperate, tropical	Climber
*Bauhinia tomentosa*	Caesalpinioideae	22/01/2017	11° 5' 34.3428" N 76° 45' 39.2688" E	8.86 ± 0.99	31	Mozamique, Zimbabwe, India and Sri Lanka	Tree
*Cassia fistula*	Caesalpinioideae	28/01/2017	11° 5' 37.9212" N 76° 46' 27.2352" E	6.63 ± 1.82	23	Indian sub-continent	Tree
*Albizia lebbeck*	Mimosoideae	04/02/2017	11° 5' 34.2852" N 76° 45' 19.2852" E	5.86 ± 1.29	4	Tropical, sub-tropical	Tree
*Acacia chundra*	Mimosoideae	06/02/2017	11° 4' 44.5548” N 76° 46' 8.7024" E	9.06 ± 0.86	8	Indian sub-continent	Tree

Seeds of each of the five species were collected directly from 16 (in the case of *Cassia fisula*) to 23 (in the case of *A. precatorius*) individuals during their natural dispersal period in 2017. To ensure fully matured and naturally dispersed seeds were collected, branches were covered with plastic bags containing holes (for air and moisture exchange) that were fixed with nylon thread and left for 11–15 days (Table 1). Seeds that fell naturally into the bags were pooled and brought back to the lab on the same day and cleaned and stored in glass jars at room temperature (20 ± 1°C; 50–60% RH) for 2 days before use in experiments. Seeds were extracted from pods by tearing them open by hand or with the aid of a scalpel. Seeds were visually inspected and insect-infected seeds were discarded.

### Moisture Content and Imbibition Testing

The moisture content of seeds of all five species was calculated by using three replicates of 15 seeds each and drying them at 103°C for 17 h (ISTA, 2013). The difference in fresh and dry weight is expressed on a percentage of fresh weight basis.

To determine the proportion of permeable and impermeable seeds in the seed lots, we carried out an imbibition test. Seeds that absorbed water were identified as non-dormant (because those with impermeable coats would not absorb water) and excluded from further studies. For imbibition test, seeds from all five species were placed on wet tissue in large plastic trays (60 cm × 50 cm × 12 cm, l × w × h) with perforated lids to minimize water evaporation yet allow gas exchange. Seeds were allowed to imbibe at 20 ± 1°C. Additional water was added when necessary. Seeds that swelled or germinated during the 28-day period were excluded from further analysis. Following the imbibition period, seeds that remained impermeable were dried on cotton towels at room temperature for 48 h and stored in glass jars at room temperature as described above for freshly collected seeds. Experiments with these seeds began within 1week after storing.

### Soil Temperature Measurements

Soil temperature at three locations within the natural dispersal shadow of mother plants was recorded using data-loggers (Rotronic, United Kingdom) for 2 years from 23 January 2017 to 23 January 2019 (see Table 1 for specific seed collection location). Thermocouples were placed at a depth of 2–4 cm, and temperature was measured at 1-h intervals. The mean highest, mean lowest, and median temperatures for each week were calculated.

### Mimicking Current and Future Warming Scenarios

We subjected seeds to three temperature regimes: current temperature (control), mild warming (+2°C), and extreme warming (+4°C). In our control treatment, soil temperatures recorded at the study site were replicated in a temperature control cabinet (Macro Scientific Works Pvt. Ltd., India) in the laboratory (precision ± 0.1°C). Rainfall patterns are also expected to change in the future. Water availability likely influences seed persistence, for example, by breaking dormancy when warm days follow heavy rainfall (i.e., wet heat; Van Klinken and Flack, 2005). However, accounting for rainfall dynamics in germination studies is problematic for several reasons. Firstly, seasonal shifts in rainfall are difficult to predict and are not yet available for our study site. Secondly, it is not known how long soils might retain moisture at elevated temperatures. In this study, we replicated the MoEF’s suggestion on rainfall changes published for Western Ghats (Rajendran et al., 2012).

Seeds were sprayed with water whenever rain fell at the collection site. The amount of water sprayed was determined by the amount of water captured by “miniature-plots” – plastic-boxes identical in size to those used in laboratory examination – filled with soil and placed on the ground. We then determined the moisture content of the soil samples and sprayed water in the trays containing seeds. In this way, our experiments enabled seeds to experience rainfall regimes akin to those occurring in the field.

Three replicates of 50 seeds per species were placed in nylon mesh bags (22 cm × 13 cm) and buried 2–4 cm deep in plastic trays (70 cm × 45 cm × 30 cm; l × w × h) filled with natural soil (sieved to remove debris). Twelve trays per species were prepared, each containing three nylon bags with 50 seeds each. Four trays per species were assigned to each temperature regime in germination chambers. For a 24-h cycle of the “current climate” chamber, we used 12 h average low temperature (7P.M.–7A.M.) followed by 4 h of median temperature (7A.M.–11A.M.) followed by 4 h of average highest temperature (11A.M.–3P.M.) and finally 4 h of median temperature (3P.M.–7P.M.). In the mild and extreme warming treatments, a similar cycle was applied, however, all temperatures were set 2 and 4°C higher, respectively. Light was provided from 7 A.M. to 7 P.M. at an intensity of 25 and 40 μmolm^−2^ s^−1^ synchronized with the median and highest average temperatures, respectively. Temperatures were adjusted weekly in accordance with data obtained from the field. After 6, 12, 18, and 24 months, one tray per species per treatment was removed, and seeds in the bags were examined. Seeds that had germinated inside the bag were counted. Germination percentage was calculated using the number of germinated seeds upon initial examination of each tray.

### Statistical Analyses

To test the effects of retrieval time, temperature regime and species on germination percentage, we performed a General Linear Model (GLM) with germination percentage as the dependent variable and retrieval time, temperature regime, and species as predictor variables. Germination percentage was converted to proportion by multiplying 0.01 and then subject to arcsine-transformation to promote normality. We used reverse stepwise model selection, whereby interactions between all predictor variables were included in the initial model and then removed in a stepwise procedure, using the lowest Akaike’s information criterion (AICc for small sample sizes) to determine the “best model” (i.e., the most parsimonious). All statistical analyses were performed in the R environment (v. 3.6.0, R Core Team, 2020), and GLMs were performed using the “lme4” package (Bates et al., 2012).

## Results

**Figure 1:**
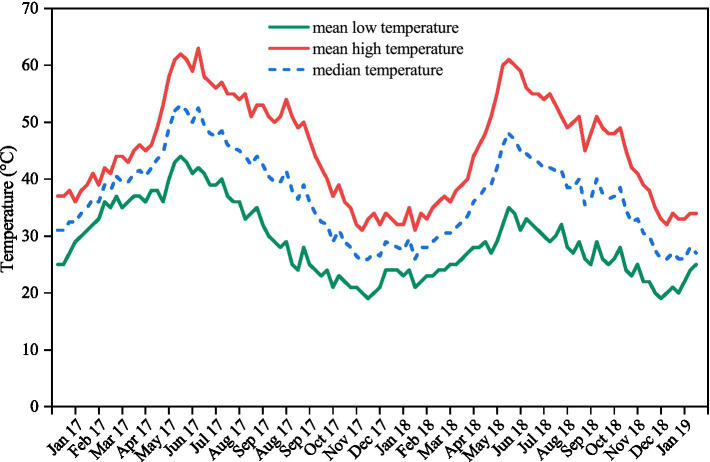
Mean high, mean low, and median weekly soil temperatures recorded in the seed collection site between 23rd January 2017 and 27th January 2019. Values are the mean of measurements made at three locations within a 2 km radius using data-loggers buried at a depth of 2–4 cm in the field.

The moisture content of the seeds of each species ranged from 5.86% for *A. lebbeck* to 9.06% for *A. chundra* (Table 1). Impermeable seeds at the time of collection varied from 4% for *A. lebbeck* to 31% for *B. tomentosa* (Table 1). Permeable seeds of most of the species swelled and germinated within 10 days after being placed on a moist substrate, although a few seeds of *A. lebbeck* did not absorb water until after 10 days. Soil temperatures peaked during the summer months (May–July) and were lowest during the winter months (Dec–Feb). Mean soil temperature ranged from 63 to 19°C (Figure 1).

### Overall Effects of Retrieval Time, Temperature Regime, and Species

**Figure 2:**
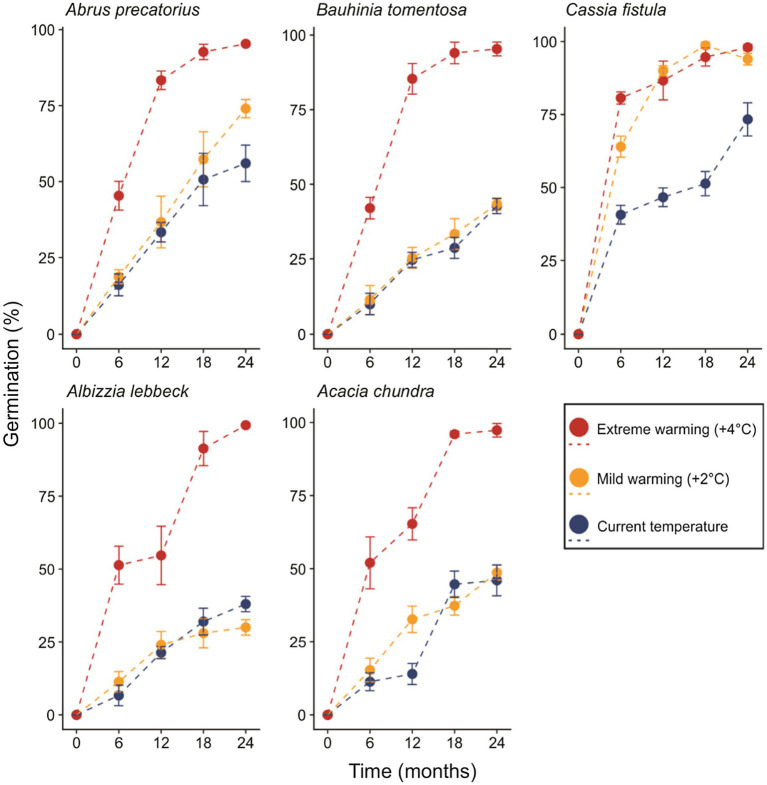
The proportion of seeds germinating over 2 years under control, mild, and extreme warming treatments. Error bars represent the SD.

In the most parsimonious generalized linear model (sin^−1^ √ [0.01 × germination percentage]~retrieval time × temperature regime + species), an interaction between the fixed factors “retrieval time” and “temperature regime” remained, while interactions of either factor with species did not (Table 2). Percent germination varied as a function of retrieval time (*T* = 2.319, *p* = 0.024, Figure 2). The effect of temperature regime was primarily seen in the significant interaction between retrieval time and temperature regime (*T* = 3.451, *p* < 0.001), indicating that the relationship between retrieval time and germination differs across temperature regimes. That is, the relationship between germination percentage and retrieval time is steeper in the warming treatments relative to control (i.e., seeds left the soil seed bank earlier, Figure 2). This relationship was also identified as significantly different in *C. fistula* (*T* = 2.780, *p* = 0.007). Curiously, *A. lebbeck* exhibited lower germination percentage relative to control in the mild warming treatment after 24 months, however, differences in the responses to control and mild warming in this species were not statistically significant. *Cassia fistula*, on the other hand, exhibited similarly increased germination percentages relative to control in both the mild and extreme warming treatments.

**Table 2:** Results of overall general linear model (GLM) on the effects of retrieval time (months), temperature regime (control, +2°C, and +4°C), species, and the interactive effect of retrieval time and temperature regime (Time: temp) on germination percentage.

	Estimate	Standard Error	*T*-value	*P*-value
Intercept	0.042	0.118	0.356	0.723
Retrieval time	0.017	0.007	2.319	0.024^*^
Temperature regime	0.076	0.050	1.511	0.135
*Acacia chundra*	−0.067	0.075	−0.919	0.361
*Albizia lebbeck*	−0.120	0.075	−1.613	0.111
*Bauhinia tomentosa*	−0.088	0.075	−1.174	0.245
*Cassia fistula*	0.207	0.075	2.780	0.007^**^
Time: temp	0.012	0.003	3.451	< 0.001^***^

*** *p* < 0.001;

** *p* < 0.01;

* *p* < 0.05.

Significant *p* values are denoted in bold.

## Discussion

The implications of climate change for the regeneration of tropical species are poorly understood, largely because of disagreement about the degree to which the tropics are expected to warm. However, there has been renewed interest in this area (Clark, 2004; Corlett, 2012; Mau et al., 2018). To understand how climate warming might affect the regeneration of tropical legumes with PY, we documented the germination dynamics of five species under two future climate scenarios using a long-term warming experiment. PY was confirmed in all five species using imbibition tests (Table 1). While PY has been confirmed in four of these species previously (Jayasuriya et al., 2013), our study appears to be the first to document PY in *A. chundra*.

Many tropical species with PY disperse their seeds at the transition of the wet to dry seasons (Vázquez-Yanes and Orozco-Segovia, 1993; Khurana and Singh, 2001). In the tropics, dormancy is broken by seasonal temperature fluctuations and the extreme soil temperatures (up to 60°C) experienced in summer. In our experiment, a proportion of seeds of all five species incubated under current climatic conditions germinated after 6 months (Figure 2). Data-loggers confirmed that soil temperature exceeded 60°C before the 6-month retrieval, suggesting that current summer temperatures break dormancy in a proportion of seeds (Figure 1). Similar patterns of dormancy break have been observed in *Dodonea viscosa* (Jaganathan and Liu, 2014), *D. hackettiana* (Cook et al., 2008), *Delonix regia* (Jaganathan et al., 2017), and *Adenanthera pavonina* (Jaganathan et al., 2018), where seeds artificially buried at a depth of 3 cm–5 cm exhibited 20–40% germination after the first summer.

Physical dormant seeds can persist in the soil for extensive periods of time (Baskin and Baskin, 2014). Indeed, most seeds in the five species we examined remained dormant after 2 years under current climatic conditions. However, the predicted warming in the tropics of 2–5°C by 2100 (Betts et al., 2011; Joshi et al., 2011; Corlett, 2012) may reduce the ability of physically dormant seeds to persist in the soil for so long. Results from our mild warming treatment (+2°C) were idiosyncratic among species. *Bauhinia tomentosa* and *A. chundra* exhibited little to no difference in germination percentages relative to control across the entire 2-year period. Seeds of *A. precatorius* also were not affected by 2°C of warming for much of the experiment, although increased germination was observed in month 24. Interestingly, *C. fistula* seeds exhibited markedly higher germination rates when incubated under both warming treatments, indicating that this species is particularly sensitive to changes in temperature (Figure 2; Table 2). Such idiosyncratic responses to climate warming might be explained by differences in the conditions required to break dormancy, such as seed moisture content (Jaganathan, 2016), seed coat thickness (Russi et al., 1992; Venier et al., 2012), and seed size (Halloran and Collins, 1974; Rodrigues-Junior et al., 2018), all of which could act independently or in tandem. Why *C. fistula* seeds were particularly sensitive to warming remains unknown and warrants further study.

In contrast to our mild warming treatment, seeds of all species subjected to our extreme warming treatment (+4°C) exhibited considerably higher germination percentages relative to control seeds (Figure 2). Cochrane (2017) observed similar results in the seeds four *Acacia* species from Australia that were incubated at 20/65°C for 112 days. Our extreme warming treatment generated very high soil temperatures during both summers (65 and 67°C, respectively), mirroring those of Cochrane (2017). Collectively, these results suggest that temperatures exceeding 65°C deplete the persistence of physically dormant seeds in the soil. Soil temperatures of 65°C are relatively common in tropical soils (Cook et al., 2008; Jaganathan and Liu, 2014), however, temperatures may reach 70°C in some open canopy areas (although direct soil temperature measurements are lacking). Thus, a threshold temperature appears to restrict the conditions under which PY can effectively operate. How exactly the warming of tropical soils beyond this threshold will affect the distribution of these species (and others) is not known.

The conditions required to break PY in the field have received some attention (Quinlivan, 1961, 1968; Taylor, 2005). Vázquez-Yanes and Orozco-Segovia (1982) demonstrated that impermeable seeds of *Heliocarpus donnell-smithii* matured in Mexico become permeable only when soil temperature exceeds 30°C. Further, more seeds become permeable to water in open canopy sites than under closed canopy, mainly because diurnal temperatures fluctuations in open canopy sites are more extreme. McDonald (2000) similarly found that subjecting seeds to varying temperature regimes of 57/23 and 70/23°C breaks PY, and that this effect was especially pronounced at high temperatures. In the future, a warmer climate is likely to bring more extreme diurnal fluctuations at higher temperatures, potentially rendering PY ineffective as a means of long-term persistence for some species in the soil.

Jaganathan (2016) previously classified PY into two levels: *shallow* and *absolute*. For seeds with shallow PY, their moisture content approaches the range at which impermeability to water is achieved (e.g., 8–10%). Seeds with a much lower moisture content of 5–8%, on the other hand, comprise the absolute PY category. We observed considerable variation in moisture content both within and among species (Table 1). Idiosyncratic responses to our warming treatments may, at least in part, be explained by this variation in moisture content. Alternatively, they may represent species-specific adaptations to certain temperature thresholds.

Taylor (1981) proposed that PY loss occurs in two-steps: (1) preconditioning, where seeds are conditioned and made sensitive, but seeds remain impermeable to water; and (2) actual dormancy-break, during which the specialized structures present in seeds (e.g., the lens in Fabaceae) opens, through which water enters and hydrates the embryo. Similar mechanisms of dormancy break have been observed in other families, such as Convolvulaceae (Jayasuriya et al., 2009) and Geraniaceae (Gama-Arachchige et al., 2012). These mechanisms are directly controlled by seasonal changes in temperature. Accordingly, we hypothesize that both stages of dormancy loss will be altered under a warmer climate. Consequently, the bet-hedging mechanism (Philippi and Seger, 1989; Ooi et al., 2009) to spread the risk of germination over time is particularly expected to be altered. One possibility is that warmer temperatures and reduced relative humidity in the future may dry seeds out more, rendering physical dormancy harder to break, and thereby compensating for higher summer temperatures (Bolingue et al., 2010). The effects of climate warming may also occur at the stage of seed maturation on the mother plant. Indeed, the effects of maternal environment on germination dynamics of PY species is gaining attention (Liyanage and Ooi, 2015; Jaganathan and Biddick, 2020), and the ultimate effect of climate warming on species with PY may result from an interplay of processes occurring both before and after dispersal. However, exactly how each stage of dormancy-break will be affected in the future requires further study.

In conclusion, it appears that an increase in temperature of 4°C will significantly increase dormancy loss and alter germination timing of species that employ PY in the tropics, potentially affecting plant establishment and community composition. A milder increase in temperature of 2°C is likely to affect fewer species than 4°C. How severely the germination ecology of species will be affected by climate warming is difficult to ascertain as responses are highly idiosyncratic.

## Data Availability Statement

The original contributions presented in the study are included in the article/supplementary material, further inquiries can be directed to the corresponding author.

## Author Contributions

GJ conceived the idea, performed the experiment, and wrote the manuscript. MB analyzed the statistics and wrote the manuscript. All authors contributed to the article and approved the submitted version.

## Funding

The financial support by National Science Foundation China (NSFC) with grant number 31750110474 is gratefully acknowledged. Funders play no role in the design of the study and publication of the results.

## Conflict of Interest

The authors declare that the research was conducted in the absence of any commercial or financial relationships that could be construed as a potential conflict of interest.

## Publisher’s Note

All claims expressed in this article are solely those of the authors and do not necessarily represent those of their affiliated organizations, or those of the publisher, the editors and the reviewers. Any product that may be evaluated in this article, or claim that may be made by its manufacturer, is not guaranteed or endorsed by the publisher.
